# Large variations in total and allele-specific transcript expression in a disease mutation-independent manner

**DOI:** 10.1038/s41598-026-40624-1

**Published:** 2026-02-27

**Authors:** Moritz Freyberg, Merete Bewig, Giovana Bavia Bampi, Candela Manfredi, Disha Joshi, Robert Rauscher, Jeong S. Hong, Jörg Große-Onnebrink, Sivagurunathan Sutharsan, Florian Stehling, Ingrid Bobis, Manfred Ballmann, Eric J. Sorscher, Zoya Ignatova

**Affiliations:** 1 Institute of Biochemistry and Molecular Biology, University of Hamburg, Hamburg, Germany; 2 Faculty of Medicine, Saarland University, Homburg, Germany; 3 Department of Pediatrics, School of Medicine, Emory University, Atlanta, USA; 4 Children’s Healthcare of Atlanta, Atlanta, USA; 5 Münster University Hospital, Münster, Germany; 6 Department of Pulmonary Medicine, Division of Cystic Fibrosis, University Medicine Essen-Ruhrlandklinik, University of Duisburg-Essen, Essen, Germany; 7 Pediatric Pulmonology University Hospital of Essen, Essen, Germany; 8 Christiane Herzog Zentrum Nord, Städtisches Krankenhaus Kiel, Kiel, Germany; 9 Pediatric and Adolescence Clinic, University Medicine Rostock, Rostock, Germany

**Keywords:** Gene expression, Allele skewing, mRNA, Monogenic diseases, Cystic fibrosis, Diseases, Genetics, Medical research, Molecular biology

## Abstract

Individuals with monogenic diseases, even those with identical disease-causing mutations, exhibit considerable clinical heterogeneity in severity and outcomes. In this study, we quantified total and allele-specific transcript levels in nasal brushings from patients diagnosed with cystic fibrosis (CF) who are homozygous or compound heterozygous for variants in the cystic fibrosis transmembrane conductance regulator (CFTR) gene. Among CF patients, total CFTR transcript levels showed greater variability than in non-CF individuals, although both groups exhibited a broad range of transcript expression. Compound heterozygous study subjects also displayed notable differences in allelic dosage, known as allelic skewing. The expression from the non-F508del allele predominated in the total transcript pool. For a small group of patients with nasal brushings available before and after treatment with FDA/EMA-approved drugs Trikafta/Kaftrio (a combination of elexacaftor-tezacaftor-ivacaftor) or Symkevi (a combination of tezacaftor-ivacaftor), we observed no effect on total CFTR transcript abundance but detected changes in allelic expression patterns. These findings reveal important aspects to be considered for personalized therapeutic approaches to CF and other monogenic diseases and emphasize previously unappreciated aspects of mRNA expression during pathogenesis.

**Supplementary Information:**

The online version contains supplementary material available at 10.1038/s41598-026-40624-1.

## Introduction

Mendelian diseases exhibit variability in clinical manifestations which complicate patient management and therapy^1,2^. Genetic modifiers, both intra- (i.e., a mutation within the disease-associated gene) and intergenic (i.e., a mutation at a different related or ostensibly unrelated locus), as well as diverse environmental and epigenetic factors^2–4^ contribute to heterogeneity in disease phenotype despite the same underlying disease-associated mutation(s). For example, epistasis can alter the magnitude of a mutational effect by influencing processes that impact disease gene expression (e.g., mRNA transcription and stability, mRNA translation, protein folding, and stability)^5–13^.

From the perspective of inherited disorders and compound heterozygous recessive genotypes, the presence of two alleles with distinct genetic abnormalities further shapes the spectrum of clinical manifestations and the breadth of outcomes. Missense mutations and even synonymous polymorphisms can have differential effects on mRNA stability^5,7,12,14^ and, consequently, alter transcript levels from the cognate allele. Nonsense mutations lead to premature termination codons (PTCs) and stimulate nonsense-mediated mRNA decay with more significant decreases of mRNA^15,16^. Furthermore, intronic variants linked to exon skipping can reduce transcript levels^17^. In such settings, although unequal allelic expression may contribute to phenotypic differences among patients and confer heterogeneity in clinical course, this possibility has not been adequately explored. In general, non-additive relationships between mutations represent less well-understood contributors to disease, clinical severity, and therapeutic responsiveness^18,19^.

In the present study, we evaluated mutations that lead to cystic fibrosis (CF)—an inherited disease associated with abnormalities of the CF transmembrane conductance regulator (CFTR)^20^—for their effects on total *CFTR* transcript expression and allele-specific dosage among compound heterozygous patients. Individuals with CF, including those with identical genotypes, exhibit a high degree of phenotypic diversity in terms of both clinical complications and overall survival^3,4^. Strikingly, dizygotic and monozygotic twins with the same genotype^21^, as well as monoamniotic monochorionic twins, i.e., the most identical sibling pairs, can show substantial variation in disease phenotype despite sharing CF-causing mutations^22^.

**Fig. 1:**
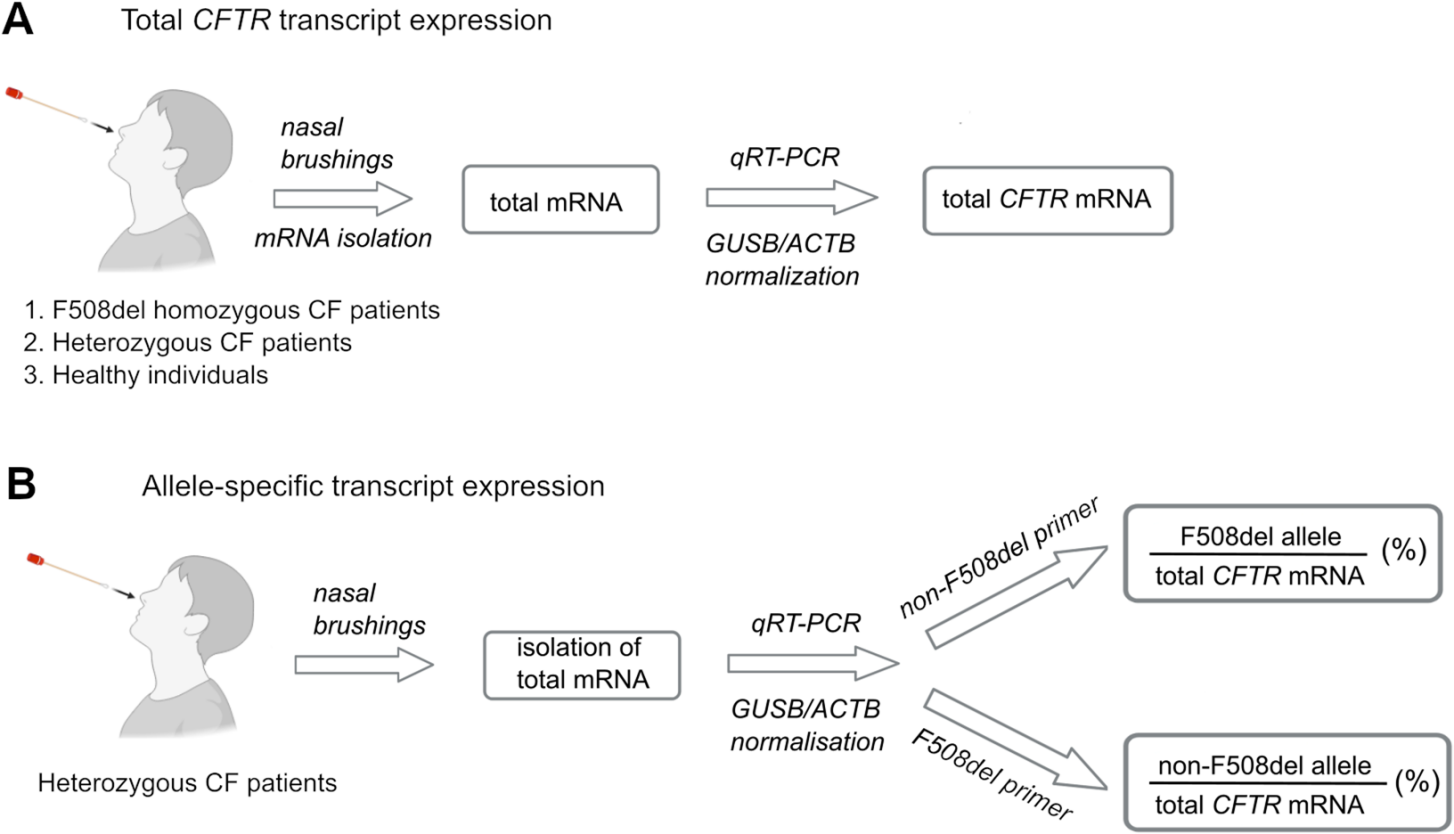
Schematic overview of our study design. (**A**) Nasal brushings from CF patients (compound heterozygous and F508del homozygous) and healthy individuals were collected and analyzed for total *CFTR* mRNA, and normalized to two housekeeping genes (*GUSB* and *ACTB*). qRT-PCR, quantitative RT-PCR. (**B**) Allele-specific transcript dosage was additionally analyzed in compound heterozygous patients with one F508del allele using primers that specifically recognize the F508del (F508del primer) and the non-F508del (non-F508del primer) alleles.

Over 2000 distinct genetic variants have been reported in the *CFTR* gene (https://cftr2.org/). Among these, more than 300 are confirmed as CF disease-causing (http://www.genet.sickkids.on.ca/). The F508del mutation is the most prevalent CF-associated defect and is observed in upwards of 85% of CF patients worldwide. Approximately 44% of patients with CF are homozygous for the F508del mutation (Cystic Fibrosis Foundation Annual Report 2021)^23,24^. Individuals with CF often display additional polymorphisms within the same allele^25^, and such mutations can differently influence mRNA stability^26^, as well as mRNA translation, protein folding, and function of CFTR^6,8–10,13,27^. Nonadditive relationship(s) between CFTR mutations further complicate in silico predictions of effects on transcript expression^10^. Here, we quantified total and allele-specific *CFTR* transcript levels in nasal brushings from CF patients with distinct genotypes (Fig. 1). Our data revealed marked variation in *CFTR* transcript levels among patients with CF, both compound heterozygous and homozygous for F508del. Allele-specific transcript-level mapping showed in compound heterozygote individuals a skewed expression pattern with higher levels of the non-F508del allele. For a small group of patients with nasal brushings available before and after treatment with Trikafta/Kaftrio (elexacaftor-tezacaftor-ivacaftor—hereafter abbreviated ETI) or Symkevi (tezacaftor-ivacaftor—abbreviated TI), we observed that treatment affected the allele-specific expression.

## Results

### *CFTR* transcript levels vary among CF patients, independent of *CFTR* genotype

We first sought to determine the overall transcript abundance in nasal brushings collected from individuals with CF (Fig. 1). Over five years, more than 200 pediatric and adult patients were enrolled in an observational study. Nasal brushings from a small number of healthy non-CF controls were also obtained. Due to bacterial infection in CF airways, associated chronic inflammation, persistent sloughing of senescent epithelial cells, and other factors that compromise RNA integrity, stringent thresholds were established for mRNA quality. Total RNA was isolated from all collected nasal brushings, but only samples with *R*NA *i*ntegrity *n*umber (RIN) ≥ 6 (see Methods for details) were examined further. The 45 high-quality samples from 30 patients (before treatment with ETI or TI) and 10 patients who had started therapy represent a sizeable group, allowing robust statistical analysis. First, we evaluated the total *CFTR* transcript levels (Fig. 1) by quantitative RT-PCR (qRT-PCR) using *CFTR* transcript-specific primers (Fig. S1). The abundance of the *CFTR* transcript was normalized to one of two reference genes: *ACTB*, a housekeeping locus commonly utilized for normalization^28–30^, or *GUSB*, which has much lower expression than *ACTB* and has been validated as a suitable reference for low-level *CFTR* mRNAs^31^.

**Fig. 2:**
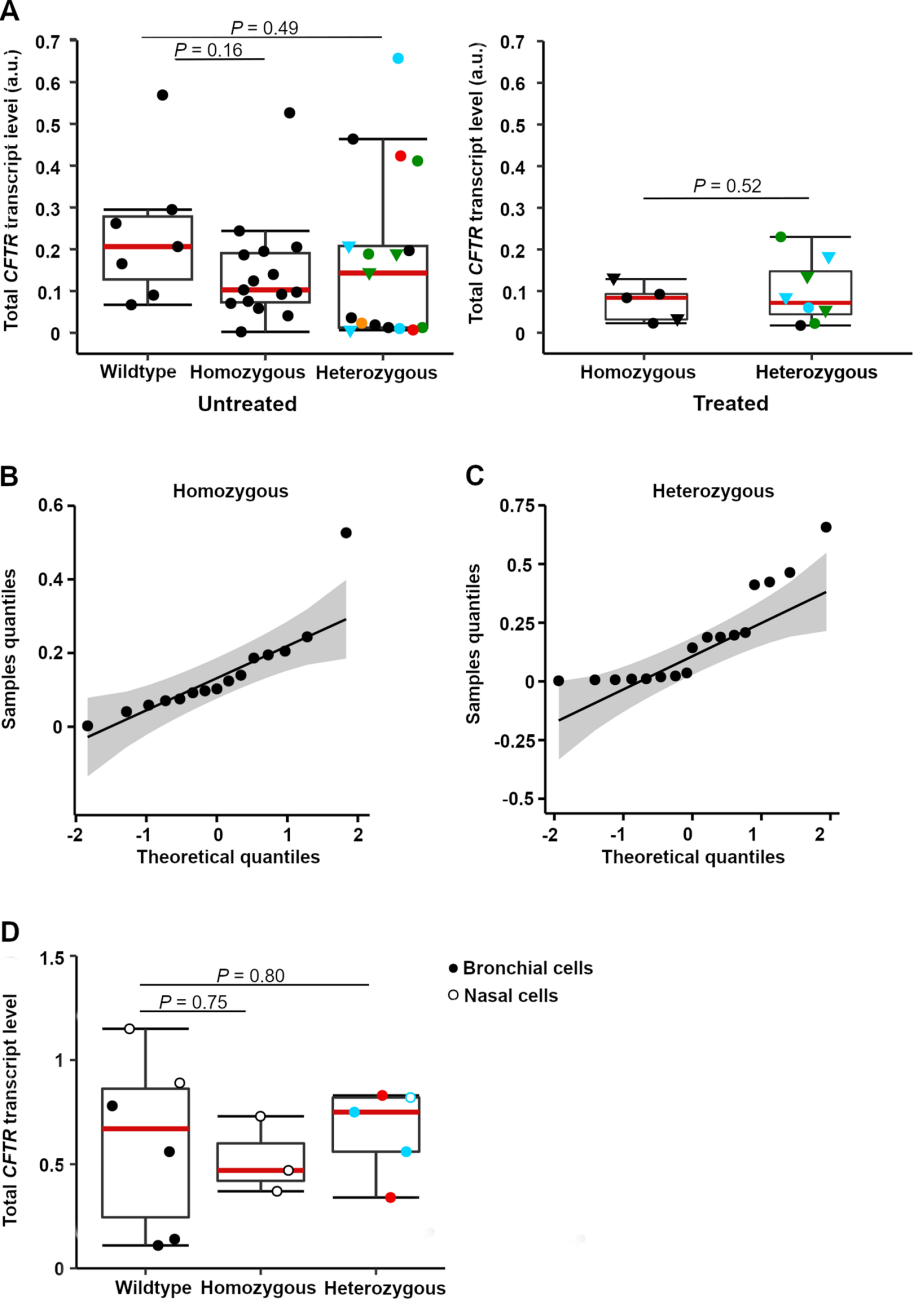
High variations in the total *CFTR* transcript levels. (**A**) Box plots of total *CFTR* transcript levels in freshly isolated hNE brushings from CF patients not treated (left plot) and treated (right plot) with CFTR modulators, as well as non-CF individuals (wildtype), analyzed by qPCR. *GUSB* mRNA was used as a reference transcript for normalization. Sizes of groups were as follows in the left plot of untreated: non-CF individuals (*n* = 7), F508del homozygous (*n* = 15 individuals; Table S1), and 15 compound heterozygous individuals (Table S2; from two patients, samples were collected at two different time points indicated by identical symbols (green and blue triangles), resulting in a tota ofl *n* = 17 samples). In the right plot patients treated with CFTR modulators: 4 individuals F508del homozygous (from one patient, sample was collected at two different time points indicated by triangles resulting in total *n* = 5 samples; Table S3), 6 individuals heterozygous (from two patients, samples were collected at two different time points indicated by identical symbol (green and blue triangles) resulting in total *n* = 8 samples; Table S4). Compound heterozygous patient mutations are color-coded: F508del compound heterozygous with missense (blue), frameshift (green), splicing/intronic (black), nonsense mutations (orange), and heterozygous with no F508del allele (red) are shown; red line, median value. Student’s t-test (two-tailed) was used to determine statistical significance. (**B**, **C**) Distribution analysis of total *CFTR* transcript values of homozygous (**B**) and compound heterozygous (**C**) patients using Q–Q plot. The confidence interval is 0.95. (**D**) Total *CFTR* transcript levels in precultured hNE (open circles) and hBE (closed circles) derived from 6 healthy individuals (wildtype), 3 homozygous (F508del) patients, and 5 compound heterozygous CF patients were normalized to *GUSB* mRNA. Compound heterozygous patient mutations are color-coded as in panel A: F508del compound heterozygous with missense (blue), and heterozygous with no F508del allele (red).

Among patients who had not received treatment, 15 (50%) were homozygous for the most common CF mutation, F508del, and 15 (50%) were compound heterozygous (13 carried the F508del mutation on one allele) (Tables S1 and S2). From two compound heterozygous patients and one homozygous individual, two samples were collected twice over a prolonged time period during which the health status was not monitored. Given the possibility that those patients may have experienced new infections or pulmonary exacerbations, they were considered independent samples (Fig. 2A). For individuals in either cohort (i.e., F508del homozygous or compound heterozygous), we observed large variations in *CFTR* transcript levels, independent of the housekeeping gene used for normalization (Figs. 2A, B, C and S2A). Some patients exhibited extremely low *CFTR* transcript levels. In the control group of non-CF individuals, *CFTR* transcripts also spanned a substantial range (Fig. 2A and S2A). The median value of the (non-CF) control group was higher than that of either CF patient cohort (i.e., homozygous or compound heterozygous). However, this difference was not statistically significant (Fig. 2A).

Among patients who have started ETI/TI therapy, four (40%) were homozygous for F508del, and six (60%) were compound heterozygous (Tables S3 and S4). Samples prior to treatment were available from four heterozygous individuals, and repeat samples were available from one homozygous and two heterozygous patients on therapy. Given the long time interval between repeat samplings and the possibility of new or recurring infections, the repeat samples were analyzed independently in the subsequent analysis. Overall, treatment with CFTR modulators did not affect the total *CFTR* transcript level (Fig. 2A right plot and Fig. S2A). We noted that following treatment with CF modulators, the variability in transcript levels decreased, i.e., mRNA levels spanned a narrower range (Fig. 1A, right plot and Fig. S2A). However, this trend might be due to the smaller cohort of ETI/TI-treated individuals.

The number of patients with overlapping genotypes was very small, thus precluding meaningful comparisons between treated and untreated groups. Moreover, in addition to genotype, several factors beyond the specific CFTR mutation (e.g., bacterial infection, chronicity of inflammation, microbial flora, etc.) may influence overall CFTR function^32–35^. To assess variations in transcript abundance in a more uniform system independent of in vivo stressors (e.g. infections), we also measured *CFTR* transcript levels in nasal epithelial (hNE) and bronchial epithelial (hBE) cells derived from patients with different CF genotypes. Samples for these studies were obtained from subjects homozygous for the F508del mutation (3 patients), compound heterozygous with one F508del allele (2 patients with F508del/N1303K and 1 patient with F508del/G551D), or with genotype p.N1303K/p.G542 × (2 patients) (Fig. 2D). Using droplet digital PCR (ddPCR) for quantification, we observed large differences in *CFTR* transcript levels between cells from individuals with the same genotype (Figs. 2D and S2B), corroborating data from hNE nasal brushings (Fig. 2A).

Variable *CFTR* mRNA, spanning several folds (Fig. 2A left), would consequently cause variations in the CFTR protein expression. A two-fold change in CFTR protein level has been reported to have significant effects on human phenotype^36–46^, supporting the potential clinical impact of the results shown here. PolyT and TG repeat variants, although outside the coding sequence, have been linked to mRNA levels and clinical phenotype. Two patients in our study with poly 7T-9T mutations exhibited very low mRNA abundance, a finding that supports earlier observations^47^. The length of the CFTR polyT tract negatively affects *CFTR* mRNA (i.e., 5T decreases *CFTR* mRNA by 10% and 7T-9T by more than 80% ^48^), leading to significant effects on the clinical phenotype^49^. Taken together, our data indicate that *CFTR* transcript levels may vary markedly among CF patients with different genotypes—including those with identical mutations. The variability in *CFTR* mRNA expression in non-CF individuals, however, suggests that these expression differences might be intrinsic to *CFTR* transcripts.

### Unequal allelic expression in compound heterozygous patients with CF

The heterozygous patient group displayed median total *CFTR* transcript levels comparable to those observed in F508del homozygous individuals (Figs. 2A and S2A). However, the heterozygous cohort exhibited somewhat greater variability, including some mutation-specific differences. We expected that mRNA levels for particular variants would be altered by established degradative pathways (e.g., nonsense-mediated decay) and by other factors that differentially affect *CFTR* transcript stability^26^. For compound heterozygous patients, we next determined allele-specific expression (Fig. 1) using primers that specifically recognize the F508del allele relative to the non-F508del allele carrying other CF mutations. The designed primers exhibited equal amplification efficiency across the distinct alleles (Fig. S1), allowing direct comparisons of mRNA dosage from each *CFTR* allele. Distinct CF mutations showed significant variation in the fraction of total mRNA expressed from each locus, a phenomenon referred to as allelic ‘skewing’ (Table 1). The mRNA transcript from the F508del allele was typically lower compared to the non-F508del locus (Table 1). In some cases, the expression of the F508del allele was substantially lower. Individuals with the same genotype (i.e., 3272–26 A-> G and poly 7T-9T) displayed similar allele expression patterns (Table 1).

**Table 1:** Unequal expression from each allele in untreated F508del-compound heterozygous patients with CF.

Non-F508del allele	Transcript level of F508del allele (a.u.)	Transcript level of non-F508del allele (a.u.)	Fraction of non-F508del transcript (%)
**2143delT** ^a^	**0.0246**	**0.1186**	**83**
**2143delT** ^a^	**0.0868**	**0.1011**	**54**
**CFTRdele 2.3**	**0.0687**	**0.1199**	**64**
**2991del32**	**0.0334**	**0.3781**	**92**
**4016insT**	**0.0006**	**0.0105**	**95**
***R117C*** ^a^	***0.0006***	***0.0097***	***94***
***R117C*** ^a^	***0.0609***	***0.1470***	***71***
***A349V***	***0.0558***	***0.6011***	***92***
***N1303K***	***0.0002***	***0.0063***	***97***
*3272–26 A > G*	*0.0005*	*0.0185*	*97*
*3272–26 A-> G*	*< 0.0001* ^*^	*0.1968*	*100*
*2789 + 5G-> A*	*0.1212*	*0.3426*	*74*
*polym7T-9T*	*0.0006*	*0.0354*	*98*
*polym7T-9T*	*< 0.0001* ^*^	*0.0126*	*100*
G542X	0.0043	0.0188	81

Transcript dosages derived from non-F508del alleles (13 individuals; from two patients, samples were collected at two different time points, hence, in total *n* = 15 samples) are represented as a percentage of the total *CFTR* transcript. Two individuals (Table S2) do not have the F508del allele and are not included here. Transcript levels were normalized to *GUSB* mRNA. Compound heterozygous patients with a genotype F508del/non-F508del are designated according to the non-F508del mutation: frameshift (bold), missense (bold italics), splicing/intronic (italics), nonsense (underlined).

^a^Samples from the same patient collected at different time points.

^*^Fluorescent threshold not reached during qPCR (i.e. Ct > 40).

To further evaluate non-stoichiometric expression in a more uniform system independent of in vivo stressors (e.g. infections), we measured allelic dosage in hNE or hBE primary cells derived from patients with CF with two of the genotypes assessed in Fig. 2D (2 individuals with F508del/G551D and 2 individuals with F508del/N1303K). ddPCR with SNP probes targeting different regions of the CFTR transcript was employed for quantitation (Table S5). In primary cells from patients with the F508del/G551D genotype, we observed modest skewing towards non-F508del mRNA (i.e. modest enhancement of the G551D allele expression; Table 2). Cells originating from patients with the F508del/N1303K genotype expressed nearly equal transcript amounts from both alleles (Table 2). It is important to note that cell culturing can be associated with signifficant changes in gene expression^50–52^. In turn, in vivo inflammation, infection, or hypoxia may alter epigenetic regulation and/or overall CFTR functional reserve^33–35,53,54^. This may explain differences in allelic skewing observed between freshly isolated patient samples and cultured primary cells. Together, the data demonstrate that among compound heterozygous patients with CF, *CFTR* alleles can produce mRNA at different non-equimolar ratios.

**Table 2:** Allele-dependent *CFTR* expression in primary hNE and hBE cells cultured from F508del-compound heterozygous patients with CF.

Non-F508del allele^a^	Fraction of the non-F508del transcript (%)	Cell type
G551D	58.3	hNE
G551D	56.5	hNE
N1303K	50.3	hNE
N1303K	50.7	hBE

The transcript dosage derived from non-F508del alleles is expressed as a percentage of the total *CFTR* transcript level.

^a^Note that hNE or hBE primary cells were derived from different individuals than those in Fig. 2D.

### CFTR modulators alter allele-specific transcript expression among compound heterozygous patients with CF

For the approved CFTR modulators ETI and TI, substantial clinical benefit among patients with CF harboring an F508del allele has been well established^55–58^. Overall, the total *CFTR* mRNA in the nasal brushings of the CF patients we analyzed did not show significant changes after treatment (Fig. 2A). We next assessed the allele skewing in the compound heterozygous patients after treatment with ETI or TI. Compared with untreated patients (Table 1), the proportion of F508del allele expression increased after drug treatment, leading to diminished allelic skewing (Table 3). It is important to note, however, that the number of patients in the modulator-treated group was small, and that effects may be restricted to particular genotypes.

**Table 3:** Unequal expression from each allele in F508del-compound heterozygous individuals treated with modulators.

Non-F508del allele	Transcript level of F508del allele (a.u.)	Transcript level of non-F508del allele (a.u.)	Fraction of the non-F508del transcript, %
**R117C**	**0.0409**	**0.0193**	**32**
**R347P** ^a^	**0.0732**	**0.1063**	**59**
**R347P** ^b^	**0.0403**	**0.0430**	**52**
***3272–26 A-> G***	***0.0151***	***0.0024***	***14***
*2143delT* ^a^	*0.0282*	*0.0242*	*46*
*2143delT* ^b^	*0.1073*	*0.0289*	*21*
*CFTRdele 2.3*	*0.0954*	*0.1347*	*59*
*F1078delT*	*0.0126*	*0.0096*	*43*

The transcript dosage derived from non-F508del alleles is represented as a percentage of the total *CFTR* transcript. Samples were collected from 6 F508del-compound heterozygous individuals (from two patients, samples were collected at two different time points, hence, in total *n* = 8 samples; Table S4). Compound heterozygous patients with a genotype F508del/non-F508del are designated according to the non-F508del mutation: missense (bold), splicing/intronic (bold italics), frameshift (italics).

^a,b^Samples from the same individual are labeled with consecutive numbers corresponding to the chronological order of sample collection.

**Fig. 3:**
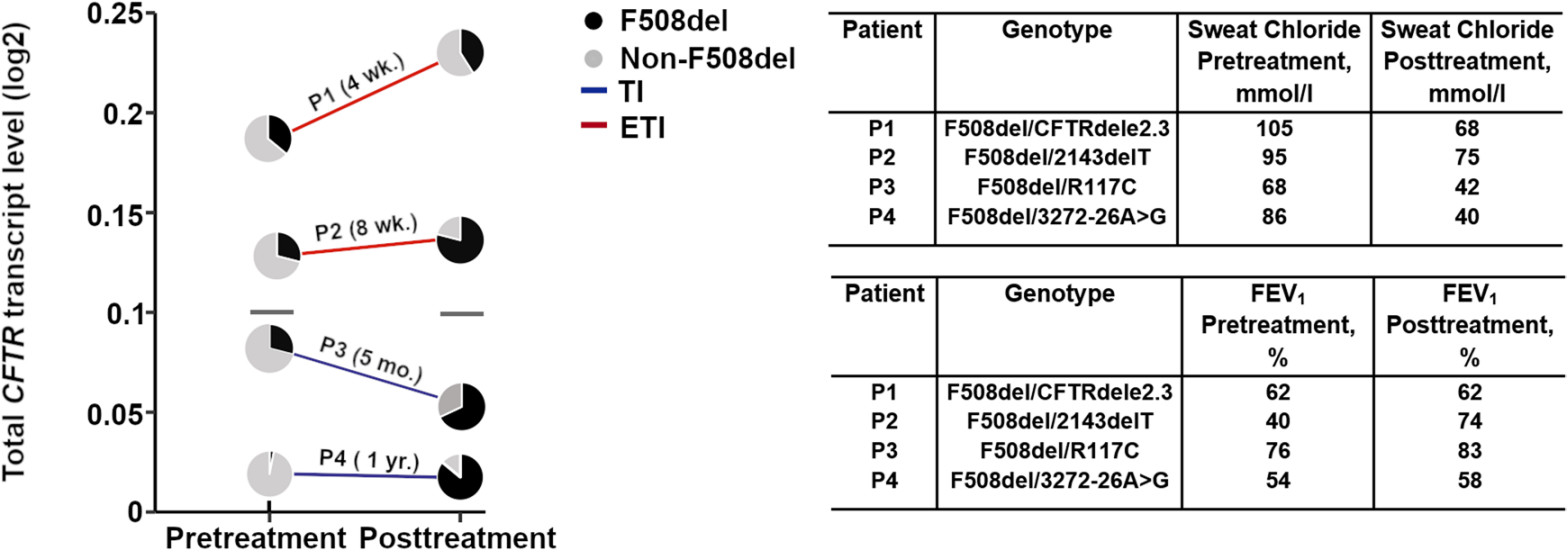
CFTR modulators enhance mRNA expression from the F508del allele. Changes in total *CFTR* transcript levels and individual allelic expression from compound heterozygous patients with CF (*n* = 4) who had brushings taken both before and after modulator treatment are shown. Two patients with 2143delT and CFTRdele2.3 (designated as P1 (F508del/CFTRdele2.3) and P2 (F508del/2143delT), respectively), were treated with ETI (red line), and two others (R117C and 3272 26 A -> C, designated as P3 (F508del/R117C) and P4 (F508del/3272 26 A -> C, respectively) received TI (blue line). The two gray horizontal lines indicate the median of total *CFTR* transcript levels. The time of treatment with the corresponding modulator is depicted; wk, week; mo., month; yr., year. Pie charts indicate the ratio of transcript level from the F508del (black) to the non-F508del (grey) allele. Table insert, changes in sweat chloride levels and FEV1 before and after treatment (see also Table S6).

For patients for whom pre- and post-modulator therapy samples were available, we compared both total and allele-specific *CFTR* transcript levels (Fig. 3). Changes in total *CFTR* mRNA following treatment were modest, with median *CFTR* mRNA in patient samples pre- and post-treatment measured as 0.105 and 0.98, respectively (i.e. not statistically different; Fig. 3, grey lines). The comparison of allelic dosage/skewing before versus after treatment suggested that modulators increased transcript levels derived from the F508del allele. In three patients, we observed a reversal of allelic skewing, with the F508del allele as the predominant source of total CFTR transcript post-treatment (Fig. 3). Observed changes in allele expression were accompanied by a substantial improvement in respiratory function (i.e., an overall increase of FEV1) and sweat chloride (Fig. 3, Table S6), as expected for patients given CFTR modulators. The result indicates that allelic skewing merits future study as a contributor to the CF clinical phenotype.

Using hBE cells derived from a patient with the genotype F508del/3272–26 A-> G, we next sought to benchmark the finding that CFTR modulator treatment increased transcript expression from the F508del allele. hBE cells were differentiated at an air-liquid interface for 21 days and treated with ETI. At 96 h post-treatment, expression from the F508del allele increased (Fig. S3A), without significant changes in total *CFTR* transcript level (Fig. S3B)—a finding consistent with our observations in nasal brushing samples (Fig. 3). Overall, these results suggest that treatment with CFTR modulators may increase transcript expression from the F508del allele.

## Discussion

We show that *CFTR* transcript levels exhibit considerable variation that cannot be predicted from specific disease-causing CFTR abnormalities. Total *CFTR* mutant transcript levels can vary by 2-5-fold, mirroring natural variations in *CFTR* expression in non-CF individuals. In compound heterozygous patients, we observed non-equimolar allele dosage skewed towards higher expression from the non-F508del allele. Despite the small patient cohort, it is reasonable to envisage that wide variations in overall *CFTR* transcript levels and transcript skewing could affect features such as clinical phenotype, prognosis, and modulator responsiveness. For example, a polyT track or TG repeat elements found in specific complex CFTR alleles can decrease mRNA abundance substantially—with both diagnostic and therapeutic implications^48,49^. Even a two-fold change in the CFTR gene product can have a substantial impact on clinical sequelae such as sinusitis, bronchiectasis and pancreatitis^39,40,44–46^.

At steady state, protein levels are assumed to be predictable from mRNA expression levels^59,60^, implying concordance between transcript levels and disease severity. Variations in transcript levels of disease-causing genes among individuals have been reported in numerous inherited conditions and have been shown to correlate with clinical outcomes^61,62^. However, transcript levels alone do not account for factors such as translation rate or mRNA utilization efficiency, post-transcriptional modifications, and post-translational regulation—features largely independent of transcript abundance that can influence steady-state CFTR biogenesis, folding, and activity^63^. In that context, CFTR undergoes complex maturational processing^27,64,65^ and distinct mutations differentially influence each of these processes. Another contribution related to variation among individuals might be a decrease in CFTR expression with age, as revealed by analyses of bulk and single-cell expression data from goblet, club, and respiratory basal cells^66^. Furthermore, recent studies provide evidence for new modes of environmental regulation of CFTR expression^67^. For example, *Streptococcus pneumoniae* infection downregulates endothelial CFTR protein expression in human lungs and is a factor that drives endothelial barrier failure in acute respiratory distress syndrome^67^. The relationship between *CFTR* transcript and protein levels is therefore highly complex and may be nonlinear—further supporting the importance of allelic skewing and other features of *CFTR* mRNA reported in this study.

Compound heterozygous patients with CF display variations in both transcript level and allele dosage. Non-equimolar allelic expression has been suggested as a hallmark of complex genomes, including both human and murine species^68,69^. Several cis- and trans-regulatory factors are believed to influence the responsible pathways^70^. For example, single-cell analysis of human primary fibroblasts has provided evidence that the majority of transcripts for most genes originate from one allele^71^. Allelic skewing during CF pathogenesis, however, has not been adequately investigated. Further studies will be needed with much larger patient cohorts and that cover the variety of CF genotypes to determine the relative contribution of each allele to total transcript levels and to correlate allele dosage with disease severity.

Combinational CFTR modulator therapy, including the approved drug combinations Trikafta/Kaftrio (ETI) and Symkevi (TI), can profoundly improve lung function and diminish pulmonary exacerbations for a sizable majority of patients with CF^72–74^—an effect conventionally attributed to correcting (elexacaftor, tezacaftor) or potentiating (ivacaftor) effects on CFTR protein folding/stability, processing to the plasma membrane, or channel function. No prior report of the influence of these treatments on CFTR transcription has been published. In a small group of patients with CF studied before and after CFTR modulation, we observed a switch in allelic skewing that alters mRNA dosage without changing the overall CFTR transcript level. The underlying molecular mechanism for this finding is unknown and will require further study of a larger number of patients with CF. Proteins have previously been shown to autoregulate their transcript abundance through various feedback loops involving specific mRNAs, thereby enhancing expression^75^. Combinatorial modulator therapy is approved for persons with at least the F508del mutation.

Along with significant improvement in CFTR protein stability and activity—effects that likely dominate the therapeutic scheme—our findings raise the possibility that functionally stabilized CFTR protein may enhance its own mRNA from the F508del allele and contribute to the palliative effects of pharmacotherapies such as ETI and TI. Such an observation might not be restricted to *CFTR* mRNA. For example, recent RNA-seq studies indicate that ETI affects mRNA expression of several other transcripts in airway epithelium. Yet it remains unclear whether the effect is direct or indirect^76^, thereby expanding the influence of ETI beyond CFTR protein folding and gating.

Notwithstanding the significance of our results, several methodological and analytical constraints warrant consideration. Our study is constrained by the relatively small number of subjects in both the CF and non-CF cohorts, limiting the generalizability of our conclusions. Moreover, the absence of detailed clinical metadata, particularly regarding respiratory infections, inflammation status, or comorbid conditions that could modulate CFTR expression, introduces potential confounders that could not be fully accounted for. In addition, sex-specific variation in CFTR transcript abundance was not assessed, as stratification by gender would have reduced the number of subjects per group and, consequently, decreased statistical power. Finally, only RNA samples with an integrity (RIN) score ≥ 6 were included to ensure high-quality transcript quantification. At the same time, this criterion enhances analytical reliability, but it may introduce technical bias by excluding specimens of lower RNA integrity. Despite these limitations, our results provide important initial insights into understanding CF genotype-phenotype correlation, the concept of missing heritability in patients with divergent clinical presentations (despite the same underlying molecular defects), and the development of personalized therapies targeting specific CF-causing variants.

## Methods

### Patient cohort and treatment

The study was approved by the Ethical Review Board of the University of Rostock (registration number: A 2016-0077). Informed consent for nasal brushings was obtained from each participant or from the participant’s parent (if the participant was a minor). Patient consent was obtained by the clinicians participating in the study and was maintained locally. All methods were performed in accordance with the relevant guidelines for the collection and analysis of patient-derived material.

Individuals with CF encoding either the F508del homozygous or a compound heterozygous genotype (with F508del) were evaluated. Non-CF volunteers were analyzed as controls. Respiratory function was measured using forced expiratory volume in 1 s (FEV1). Legacy names for mutations were derived from the CF mutation database (CFTR1: http://www.genet.sickkids.on.ca/cftr) using + 1 as the first nucleotide of the reference sequence (GeneBank NM_000492.3). Mutation nomenclature was assigned according to international recommendations (www.hgvs.org/mutnomen), i.e., using + 1 as the A of the ATG start codon in the reference, as indicated in brackets. For clarity, both the legacy name and identification are provided according to the Human Genome Variation Society (HGVS) standards.

Before therapy with ETI, patients were treated with standard supportive measures, inclduing physiotherapy, vitamins, minerals, pancreatic digestive enzymes, inhalation of hypertonic saline solution, bronchodilators, steroids, and/or antibiotics. Patients given ETI received approved daily dosages of 150 mg ivacaftor, 100 mg tezacaftor, and 200 mg elexacaftor in the morning and 150 mg ivacaftor in the evening. Each dose was taken with a fat-containing meal. One patient received a reduced dosage of 75 mg ivacaftor, 50 mg tezacaftor, and 100 mg elexacaftor once a day due to liver dysfunction. Patients who received TI treatment were administered 150 mg ivacaftor daily, 100 mg tezacaftor in the morning, and 150 mg ivacaftor in the evening.

### Sex as a biological variable

Our cohort comprises male and female human individuals. Sex was not considered a a biological variable.

### Nasal epithelial (NE) cell collection

One or two nasal brushes (Cytobrush Plus GT Sterile, Medesign #MED1000009) per patient were introduced along the septal aspect of the nostril until reaching the nasal turbinate and then repeatedly moved up and down (4-6x) while rotating 1-2x full turns. Brushes were placed in a 1.5 ml Eppendorf tube containing 1 ml of TRIzol Reagent (Thermo Scientific), flash-frozen, and kept at -80 °C until further analysis. Note that TriReagent inactivates RNases and preserves mRNA intact.

### RNA extraction and cDNA synthesis for RT-qPCR

Total RNA was extracted from NE brushings with TRIzol Reagent according to the manufacturer’s protocol (Invitrogen). RNA quality, integrity, and concentration were assessed using Bioanalyzer (Agilent), which calculates RNA integrity number (RIN). RNA samples with RIN values between 1 and 5 reflect poor-quality preparations and a significant fraction of degraded mRNA^77^, and were deemed unsuitable for further analysis. We considered samples with RIN values ≥ 6 for analysis, indicating high-quality mRNA. These samples were subsequently subjected to cDNA synthesis. Approximately 1 µg of total RNA was treated with DNase (1 U DNase/µg RNA; Thermo Scientific) at 37 °C for 1 h and subsequently underwent reverse transcription for 1 h at 42 °C using oligo(dT) primers and Revert Aid Minus Reverse Transcriptase (Thermo Scientific). Reverse transcriptase was then deactivated by adding EDTA and heating at 70 °C for 10 min.

### qRT-PCR

To ensure comparability, measurements of total and allele-specific transcript levels were both conducted using the same two primer sets (Fig. S1A–C and Table S7). One set was designed to amplify a 204-nt DNA segment containing the wild-type F508 triplet (non-F508del-specific primer). The primer set specific for F508del generated a 201-nt fragment without the three deleted nucleotides (F508del-specific primer) (Fig. S1A–C and Table S7). The same reverse primer was used in both primer sets. All samples were analyzed separately by qRT-PCR in triplicate (or duplicate when input material was limited). Considerable care was taken to develop five-point curves from a 10-fold serial dilution of a control pool sample, which was used to build calibration graphs for each PCR reaction. For all calibration curves, a correlation coefficient (R^2^) was calculated and used to assess the quality of fit. The efficiency of each primer set was calculated using E = 10(-1/slope), where E represents PCR efficiency, and “slope” is the slope of the regression curve fit. An E value of 2 corresponded to maximum efficiency. Raw quantification threshold cycle (Ct) values were determined using QPCR-Soft 3.1 (Analytik Jena). If the threshold was not reached during qPCR, the Ct value was set to 40 (i.e., the maximum number of qPCR cycles).

For control samples and compound heterozygous patients without the F508del allele, total transcript levels were determined using the non-F508del-specific primer as the forward primer. In F508del homozygous patients, the total transcript levels were measured using the F508del-specific primer as the forward primer. *CFTR* allele-specific transcript abundance was normalized (ΔCt) to the reference genes β-Glucuronidase *(GUSB)* or β-actin (*ACTB*), and mRNA levels were expressed as 2^− ΔCt^.

In compound heterozygous patients with one F508del allele, we employed both forward primers (non-F508del-primer and F508del-primer) to simultaneously determine allele-specific expression. The level of each allele was normalized (ΔCt) to the reference gene β-Glucuronidase *(GUSB)* or β-actin (*ACTB*), and mRNA levels were expressed as 2^− ΔCt^. Total expression was taken as the sum of both alleles.

### Primary cell expansion, differentiation and modulator treatment

Primary hBE cells were obtained from the Cystic Fibrosis Foundation Laboratory (Lexington, Massachusetts). 1 × 10^6^ cells were seeded onto T75 cell culture flasks coated with human type IV collagen (Sigma-Aldrich). Cells were expanded in PneumaCult-Ex Plus media (STEMCELL Technologies) supplemented with 2 µg/ml amphotericin B (Sigma-Aldrich), 100 µg/ml ceftazidime (Sigma-Aldrich), 100 µg/ml vancomycin (Sigma-Aldrich), and 100 µg/ml tobramycin (Sigma-Aldrich) for 5 days. Cells were subsequently expanded without antibiotics until 70% confluency was reached.

1 × 10^5^ expanded hBE cells were transferred to a human type IV collagen-coated permeable polyester Transwell insert (Corning). PneumaCult-Ex Plus media was added to both apical and basal compartments and replaced daily. On day 4, media was removed, and only the basal compartment was replenished with PneumaCult–ALI media (STEMCELL Technologies). Cells were differentiated for 22 days before modulator treatment.

Ivacaftor (Selleck Chemicals), tezacaftor (Selleck Chemicals), and elexacaftor (Selleck Chemicals) were combined in DMSO. Cells were treated by adding DMSO (vehicle) or ETI (5 µM for each component) in PneumaCult–ALI media to the apical membranes of cell monolayers for up to 96 h, with drug-media mix renewed after 48 h. Following treatment, cells were subjected to RNA extraction with TRIzol Reagent, and CFTR mRNA was analyzed by RT-qPCR.

### Airway epithelial culture and droplet digital PCR (dd-PCR)

Human bronchial or nasal epithelial cells (hBE or hNE) were seeded in T75 flasks coated with Purecol (Advance Biomatrix) in pre-warmed complete Pneumacult Ex Plus (StemCell Technologies, Cat. No. 05040) at 37 °C in 5% CO_2_ and expanded until reaching 70–80% confluency. When confluent, cells were detached using ACF enzymatic dissociation solution (StemCell Technologies, Cat. No. 05426) and seeded at a density of 1.5 × 10^5^ cells per insert onto collagen IV-coated 6.5 mm transwells (Costar 3470) with Complete Pneumacult Ex-Plus medium. Plates were incubated at 37 °C with 5% CO2. Media was changed daily until monolayers reached confluency, typically between days 3 and 5 after seeding. Ex Plus medium was then aspirated from both the apical and basolateral surfaces and the lower chamber fluid was replaced with airway-liquid interface (ALI) medium (StemCell Technologies, Cat. No. 05001). These conditions were maintained for 21 to 28 days until monolayers became fully differentiated.

*CFTR* mRNA measurement was performed by ddPCR using a QX200 droplet reader with an automated droplet generator. Briefly, RNA was extracted using the RNAeasy Mini Kit (Qiagen) according to the manufacturer’s instructions. RNA samples were reverse-transcribed using SuperScript IV VILO Master Mix (ThermoFisher Scientific), and the resulting cDNA products (equivalent to ~ 20 ng total RNA) were used to quantify CFTR and control genes. To measure CFTR transcript, we employed a predesigned probe specific for CFTR (FAM-labeled; assay ID: dHsaCPE5056656, Biorad) multiplexed with GUSB (HEX labeled; assay ID: dHsaCPE5050189) or TBP (HEX labeled; assay ID: dHsaCPE5058363) probes; GUSB or TBP were included as references.

Following droplet generation and PCR amplification, reactions were evaluated with a QX200 Reader (Bio-Rad). QuantaSoft Software (Bio-Rad) was used to analyze the positive and negative droplets for each fluorophore across samples, yielding the concentrations of target and reference DNA molecules expressed as copies per microliter (copies/µl) of input. Results were presented as the ratio of target gene copies to the reference gene.

### Statistical analyses

Statistical analyses were performed in R version 4.0.4 (R Core Team). Two-tailed Students’ t-test and Pearson’s correlation coefficient were used to calculate p-values and correlation, respectively. Two individuals were sampled at two different time points. Given the typically long time interval between samplings and the possibility that patients may have experienced or acquired infections in the interim, we treat the two samples from each individual as independent.

## Supplementary Information

Below is the link to the electronic supplementary material.

Supplementary Material 1

## Data Availability

All data used are available within this paper, Supplementary Information and source file. Further information can be acquired from the corresponding authors upon request.
