# Supplementary material for: Large variations in total and allele-specific transcript expression in a disease mutation-independent manner
Source: Sci Rep. 2026 Feb 27;16:7831. doi: 10.1038/s41598-026-40624-1 (PMC12953693; doi:10.1038/s41598-026-40624-1)
Supplement: Supplementary file 1 — Supplementary Material 1 [file 41598_2026_40624_MOESM1_ESM.docx]

**Supplementary Information**

**Large variations in total and allele-specific transcript expression in a disease mutation-independent manner**

Moritz Freyberg, Merete Bewig, Giovana Bavia Bampi, Candela Manfredi, Disha Joshi, Robert Rauscher, Jeong Hong, Jörg Große-Onnebrink, Sivagurunathan Sutharsan, Florian Stehling, Ingrid Bobis, Manfred Ballmann, Eric J. Sorscher, Zoya Ignatova

**
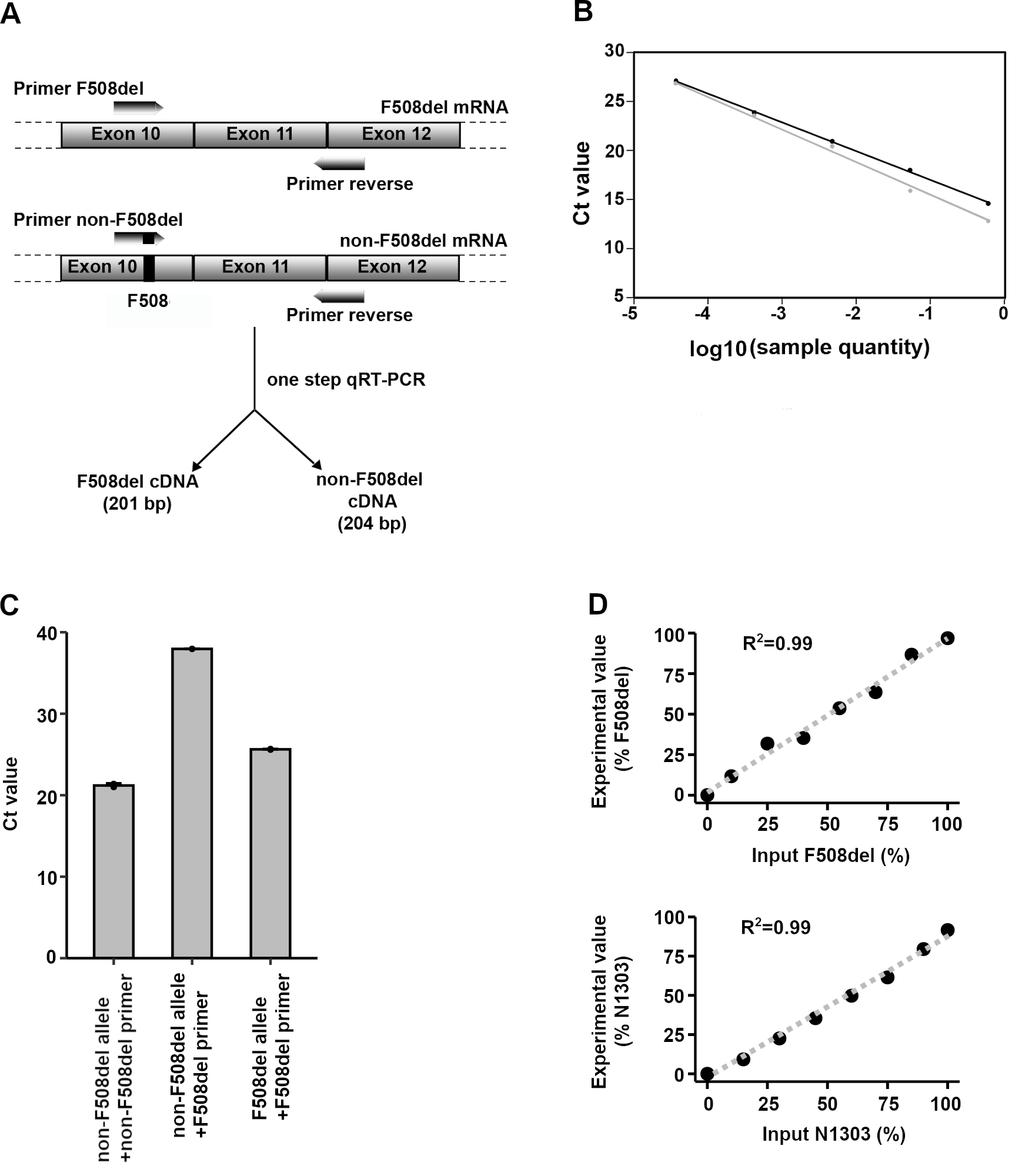
**

**Figure S1**. **Approach to determine total and allele-specific expression by qRT-PCR.** (A) qRT-PCR primers were designed to discriminate between mRNA expressed from the F508del and non-F508del *CFTR* alleles. One forward primer (F508del) bears the F508del mutation and anneals perfectly to the F508del codon and its vicinity (black bar) and thus is specific for the F508del allele (c.1521_1523delCTT); another forward primer (non-F508del) pairs in the same region, but to the wildtype *CFTR* allele and is used for amplification alleles which carry another CF mutation elsewhere in the gene but an intact F508 codon. The reverse primer is the same, binds at the exon 11/12 junction and was used in combination with either of the forward primers. For details, on transcript-level calculations, see Materials and Methods. (B) Two different alleles were amplified with the same efficiency. Regression analysis for each standard curve obtained with the non-F508del allele (black) or F508del allele (gray) using non-F508del and F508del primers (A), respectively. (C) Determination of the binding efficiency of the respective primer pairs to the corresponding match or mismatch allele. High Ct values reported on low efficiency (e.g. when using a non-matching non-F508del forward primer on the F508del allele indicate poor binding. Conversely, low Ct values report efficient binding (e.g. when using a non-F508del matching primer on a non-F508del allele or an F508del matching primer on the F508del allele). The non-F508del primer efficiently amplifies wildtype *CFTR* (i.e. low Ct value). Primer efficiency was evaluated using plasmids expressing F508del-CFTR (F508del allele) and wildtype CFTR (non-F508del allele). (D) Validation of predesigned Taqman SNP probe sets for quantitation of allele-specific transcript by ddPCR using an N1303K/F508del as a heterozygous example.

**
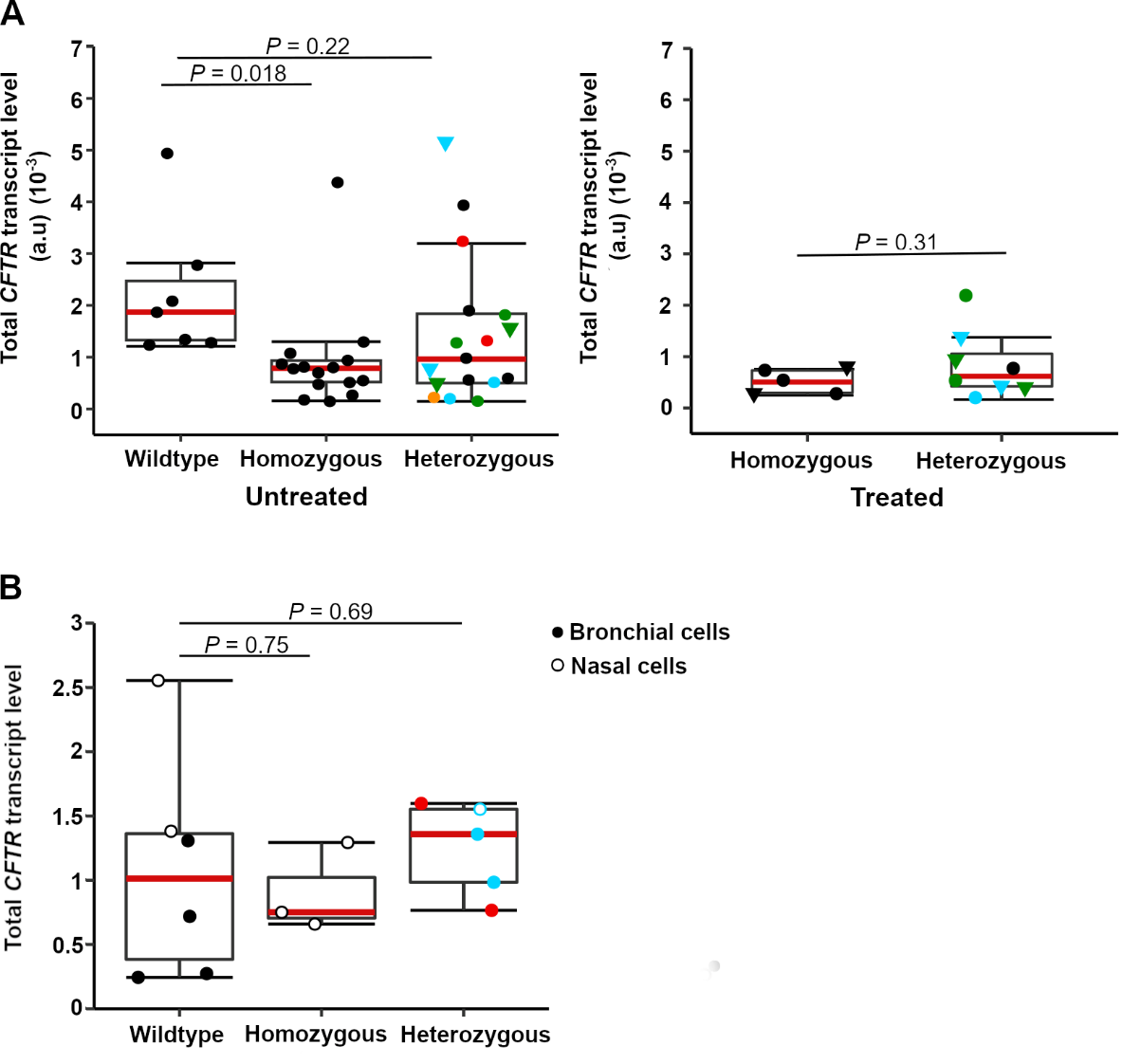
**

**Figure S2.** **Variation of *CFTR* transcript levels in CF patients and non-CF individuals.** (A) Box plot of total *CFTR* transcript in nasal brushings from CF patients not treated or treated with CFTR modulators and non-CF individuals (wildtype) analyzed by qPCR. The same samples were evaluated as in Figure 1A, but using *ACTB* mRNA as the normalization reference. Sizes of groups were as follows in the left plot of untreated: non-CF individuals (*n*=7), F508del homozygous (*n*=15 individuals; Table S1), and 15 compound heterozygous individuals (Table S2) (from two patients, samples were collected at two different time points indicated by green and blue triangles, resulting in a total of *n*=17 samples). In the right plot patients treated with CFTR modulators: 4 individuals F508del homozygous (from one patient, sample was collected at two different time points designated by triangles, resulting in total *n*=5 samples; Table S3), 6 individuals heterozygous (from two patients, samples were collected at two different time points designated by green and blue triangles, resulting in total *n*=8 samples; Table S4). Compound heterozygous patients are color-coded by the non-F508del mutation: missense (blue), frameshift (green), splicing/intronic (black), nonsense mutation (orange) and heterozygous with no F508del allele (red). Red line, median value. Student’s t-test (two-tailed) was used to determine statistical significance. (B) Total C*FTR* transcript levels in precultured hNE or hBE cells derived from 6 healthy individuals (wildtype), 3 homozygous (F508del) patients, and 5 compound heterozygous CF patients. The same samples were analyzed as in Figure 2, but were normalized to TATA-binding protein (*TBP* mRNA) as a housekeeping gene. Compound heterozygous patient mutations are color-coded as in panel A: F508del compound heterozygous with missense (blue), and heterozygous with no F508del allele (red)

**
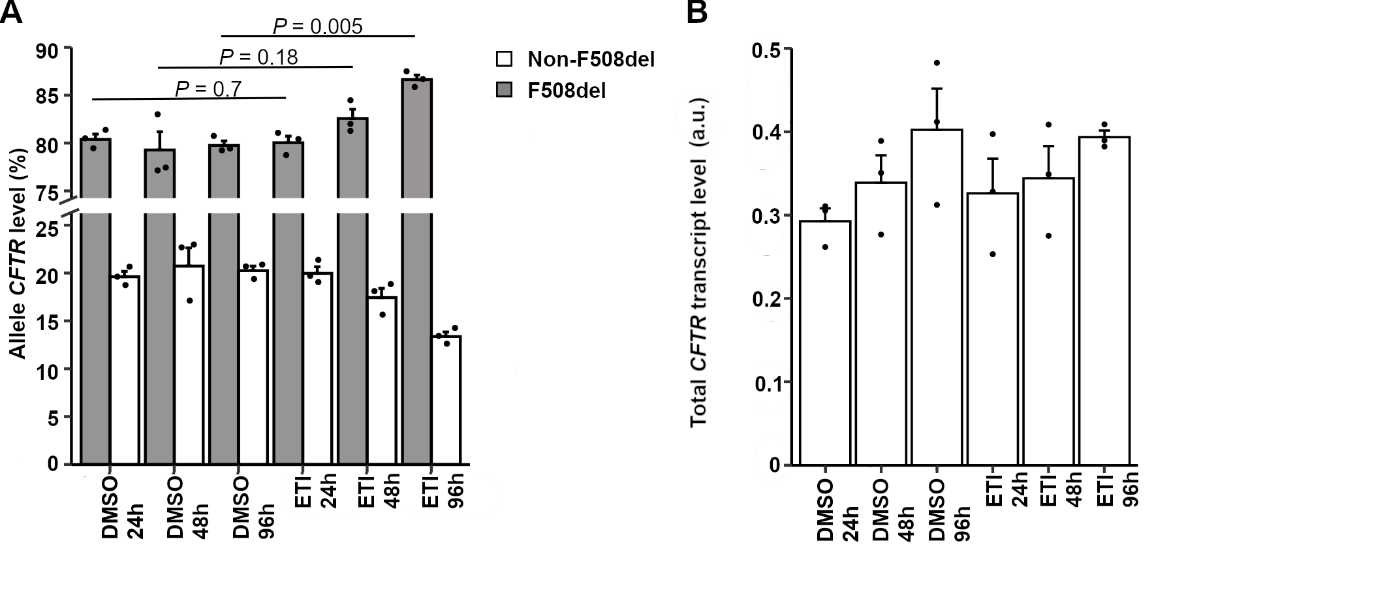
**

**Figure S3**. **CFTR modulators elevate transcript levels from the F508del allele in primary patient-derived hBE cells (genotype F508del/3272-26A->G).** (A) Changes in *CFTR* transcript dosage from F508del and non-F508del alleles over time following short-term ETI treatment are shown. DMSO was used as a vehicle control. Note the high expression of the F508del allele in ETI untreated cells Information regarding therapy of the donor with modulators prior to obtaining cells (as a possible contributor to these findings) is not available. Data are means ± SEM (*n*=3). Student’s t-test (two-tailed) was used to determine statistical significance. (B) Total *CFTR* transcript levels following treatment (*n*=3).

**Table S1. Key clinical characteristics of untreated F508del homozygous patients in the study.** One patient from the F508del homozygous cohort had no clinical data available and was therefore not displayed in the table, but is included in Figure 1A. PI, exocrine pancreatic insufficient.

| **Age (years)** | **Pancreatic status** |
| --- | --- |
| 1 | PI |
| 3 | PI |
| 6 | PI |
| 11 | PI |
| 19 | PI |
| 21 | PI |
| 21 | PI |
| 22 | PI |
| 28 | PI |
| 35 | PI |
| 41 | PI |
| 42 | PI |
| 45 | PI |
| 48 | PI |

**Table S2. Key clinical characteristics of the untreated heterozygous CF patients in the study.** Color coding based on the non-F508del mutation: missense (blue), frameshift (green), splicing/intronic (black), nonsense (orange), and heterozygous with a non-F508del allele (red). PI, exocrine pancreatic insufficient; PS, pancreatic sufficient; n/a, not assessed.

| **Genotype** | **Age (years)** | **Pancreatic status** |
| --- | --- | --- |
| F508del/R117C | 34 | n/a |
| F508del/A349V | 50 | PS |
| F508del/N1303K | 18 | PI |
| F508del/3272-26A->G | 19 | PS |
| F508del/3272-26A>G | 38 | n/a |
| F508del/2789+5G->A | 52 | PS |
| F508del/polym7T-9T | 10 | PI |
| F508del/polym7T-9T | 14 | PI |
| F508del/4016insT | 5 | PI |
| F508del/2991del32 | 18 | PI |
| F508del/2143delT | 27 | PI |
| F508del/CFTRdel2.3 | 46 | PI |
| F508del/G542X | 9 | PI |
| L139ins/del Exon 13 and 14^a^ | n/a | n/a |
| G551D/3667ins4^a^ | 22 | PI |

^a^Patients without the F508del allele are not included in Table 1.

**Table S3. Pancreatic status of F508del homozygous patients that received modulator treatment in the study.** PI, exocrine pancreatic insufficient.

| **Age (years)** | **Pancreatic status** |
| --- | --- |
| 25 | PI |
| 31 | PI |
| 19 | PI |
| 26 | PI |

**Table S4. Pancreatic status of the treated heterozygous CF patients in the study.** Color coding based on the non-F508del mutation: missense (blue), splicing/intronic (black), and frameshift (green). PI, exocrine pancreatic insufficient; PS, pancreatic sufficient.

| **Genotype** | **Age (years)** | **Pancreatic status** |
| --- | --- | --- |
| F508del/R117C | 34 | PS |
| F508del/R347P | 33 | PS |
| F508del/3272-26A>G | 39 | PS |
| F508del/2143delT | 27 | PI |
| F508del/CFTRdel2.3 | 46 | PI |
| F508del/F1078delT | 21 | PI |

**Table S5. Probe sets for quantification of total and allele-specific *CFTR* transcript levels used during ddPCR.** Total CFTR transcript level was quantified using a predesigned probe set targeting the exon 21-22 junction. Allele-specific quantification was performed using predesigned Taqman SNP (single nucleotide polymorphism) probes.

| Genes or alleles | Assay ID | Notes |
| --- | --- | --- |
| CFTR | dHsaCPE5056656 (FAM labelled; Biorad) | Total CFTR mRNA measurement (anneals at exon 21-22 junction) |
| GUSB (Glucuronidase beta) | dHsaCPE5050189  (HEX labelled, Biorad) | Total GUSB mRNA measurement |
| TBP (TATA-binding protein) | dHsaCPE5058363  (HEX labelled, Biorad) | Total TBP mRNA measurement |
| F508del (rs113993960) | C_151693869_10(Applied Biosystems, Thermo)  supplemented with a reverse primer to enable amplification of cDNA.  ΔF508_mRNA_R3 = TGTGATTCCACCTTCTCCAA | F508 or F508del allele-specific quantification of CFTR mRNA at exon 11 |
| N1303K (rs80034486) | C__32544994_20  (Applied Biosystems, Thermo) | N1303 or K1303 allele-specific quantification of CFTR mRNA at exon 24 |

**Table S6. FEV_1_ values for four patients before and after combination modulator therapy.** FEV_1_ values measured on different days for each patient pre- or post-treatment with TI or ETI. The FEV_1_ values in bold are displayed in Figure 2 and were measured on the same day as the collection of nasal brushing.

| **Patient**  **genotype** | **Pre-treatment** | | **Post-treatment** | |
| --- | --- | --- | --- | --- |
|  | **Date of measurement** | **FEV_1_ (%)** | **Date of measurement** | **FEV_1_ (%)** |
| P1  F508del/CFTRdele2.3 | May 2020  July 2020  **Aug. 2020**  Sept. 2020 | 66  64  **62**  62 | Dec. 2020  **Jan. 2021** | 65  **62** |
| P2  F508del/2143delT | **May 2020**  July 2020  Aug. 2020  Sept. 2020 | **40**  38  38  36 | Oct. 2020  Nov. 2020  **Dec. 2020** | 72  67  **74** |
| P3  F508del/R117C | Apr. 2020  **Sept. 2020**  Oct. 2020  Nov. 2020 | 84  **76**  64  78 | Dec. 2020  Jan. 2021  Feb. 2021  **Mar. 2021** | 84  81  85  **83** |
| P4  F508del/3272-26A>G | Nov. 2018  Jan. 2019  **Mar. 2019**  June 2019  Nov. 2019 | 49  57  **54**  56  57 | Dec. 2019  Feb. 2020  June 2020  July 2020  Sept. 2020  **Dec. 2020** | 59  55  54  61  60  **58** |

**Table S7. Primer sets for total CFTR and allele-specific transcript amplification used in the qRT-PCR reaction.** The forward primer aligns to the F508del or wildtype *CFTR* sequence. The reverse primer (CFTR-rev_comm) was used with either the CFTR forward primer. The sizes of the produced amplicons are displayed in Figure S1A.

| Primer Name | Primer sequence (5´-3´) | Notes |
| --- | --- | --- |
| non-F508del_forward | CTGGCACCATTAAAGAAAATATCAT**CTT** | Binds to F508 codon; used for allele-specific amplification of non-F508del containing exon |
| F508del_forward | CTGGCACCATTAAAGAAAATATCATTG | Skips F508 codon; used for allele-specific amplification of F508del containing exon |
| CFTR_rev_comm | CAGCATCTTTGTATACTGCTCTTGC | Used for amplification of total *CFTR* mRNA |
| GUSB_forward | GACACGCTAGAGCATGAGGG | Primer set for amplification of *GUSB* mRNA |
| GUBS_reverse | GGGTGAGTGTGTTGTTGATGG |  |
| ACTB_forward | CCCCAAAGTTCACAATGTG | Primer set for amplification of actin mRNA |
| ACTB_reverse | AGCAATGCTATCACCTCC |  |
